# MobiLipid: A Tool for Enhancing CCS Quality Control of Ion Mobility–Mass Spectrometry Lipidomics by Internal Standardization

**DOI:** 10.1021/acs.analchem.4c01253

**Published:** 2024-05-02

**Authors:** Felina Hildebrand, Gunda Koellensperger, Tim Causon

**Affiliations:** † Department of Analytical Chemistry, Faculty of Chemistry, University of Vienna, Waehringer Str. 38, 1090 Vienna, Austria; ‡ Vienna Doctoral School in Chemistry (DoSChem), University of Vienna, Waehringer Str. 42, 1090 Vienna, Austria; § Vienna Metabolomics Center (VIME), University of Vienna, Althanstr. 14, 1090 Vienna, Austria; ∥ BOKU University, Department of Chemistry, Institute of Analytical Chemistry, Muthgasse 18, 1190 Vienna, Austria

## Abstract

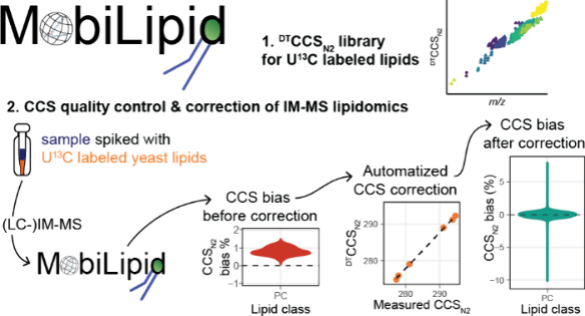

Ion mobility–mass spectrometry (IM-MS) offers benefits for lipidomics by obtaining IM-derived collision cross sections (CCS), a conditional property of an ion that can enhance lipid identification. While drift tube (DT) IM-MS retains a direct link to the primary experimental method to derive CCS values, other IM technologies rely solely on external CCS calibration, posing challenges due to dissimilar chemical properties between lipids and calibrants. To address this, we introduce MobiLipid, a novel tool facilitating the CCS quality control of IM-MS lipidomics workflows by internal standardization. MobiLipid utilizes a newly established ^DT^CCS_N2_ library for uniformly (U)^13^C-labeled lipids, derived from a U^13^C-labeled yeast extract, containing 377 ^DT^CCS_N2_ values. This automated open-source R Markdown tool enables internal monitoring and straightforward compensation for CCS_N2_ biases. It supports lipid class- and adduct-specific CCS corrections, requiring only three U^13^C-labeled lipids per lipid class-adduct combination across 10 lipid classes without requiring additional external measurements. The applicability of MobiLipid is demonstrated for trapped IM (TIM)-MS measurements of an unlabeled yeast extract spiked with U^13^C-labeled lipids. Monitoring the CCS_N2_ biases of ^TIM^CCS_N2_ values compared to ^DT^CCS_N2_ library entries utilizing MobiLipid resulted in mean absolute biases of 0.78% and 0.33% in positive and negative ionization mode, respectively. By applying the CCS correction integrated into the tool for the exemplary data set, the mean absolute CCS_N2_ biases of 10 lipid classes could be reduced to approximately 0%.

## Introduction

Integrating ion mobility (IM) technologies into mass spectrometry (MS) and LC-MS experiments offers distinct advantages over conventional MS-based lipidomics workflows. IM separation facilitates the conformational separation of lipids from other sample components and background noise, thereby leading to cleaner MS spectra. Furthermore, IM separation can support lipid annotation, as it provides a rapid confirmatory separation dimension that can be used for IM alignment of precursors and fragments in typical lipidomics workflows based on MS2. Additionally, three major commercial IM technologies (i.e., drift tube ion mobility (DTIM), trapped ion mobility (TIM), and traveling wave ion mobility (TWIM)) provide IM-derived collision cross sections (CCS) which are a conditional physicochemical descriptor reflecting the chemical structure and 3D conformation of an ionized lipid.^1−3^ Since 2014, several studies have demonstrated good interlab reproducibility^4−9^ of CCS values for lipidomics and other related applications, but most studies have been constrained to a single IM-MS technology. Moreover, the uncertainty of CCS determination remains as a major obstacle due to the lack of reference materials and the prediction of CCS values based on first principle calculations.^10,11^

When characterizing materials in regard to the CCS value of analytes, DTIM-MS remains a key reference technology, as low-field conditions are closest to fundamental IM theory, and CCS values derived from DTIM-MS have been made traceable to the primary experimental IM-MS method (i.e., stepped-field) performed on a reference instrument with highly characterized length, temperature, voltages, and pressures.^6^ All other IM-MS technologies, including TWIM-MS and TIM-MS, rely solely on external CCS calibration, with calibrants established by DTIM-MS.^11^ However, CCS calibrants usually have dissimilar chemical properties to analytes of interest, which poses some challenges for routine “omics” workflows, including lipidomics. Especially in the case of TWIM-MS lipidomics applications, it has been shown that either a calibration^12,13^ or an external postcalibration correction^14^ using (class-specific) lipids leads to an improved trueness of CCS values compared to ^DT^CCS and/or published ^TW^CCS values. Moreover, high-resolution IM analyzers, including structures for lossless ion manipulation (SLIM)^15^ and cyclic IMS^16^ offer new possibilities for challenging lipid isomer separations for which a CCS calibration provides new challenges. Recent studies revealed that ^TIM^CCS values of lipids generally show good agreement with ^DT^CCS when relying on external calibration using the same calibrant ions (ESI Tune Mix calibration standard).^9,17^ However, a CCS recalibration of each analytical run is recommended in this type of workflow. Beyond lipidomics, one recent comprehensive study compared ^TW^CCS_N2_, ^TIM^CCS_N2_, and ^DT^CCS_N2_ values of 87 steroids following the vendor-recommended calibration practices of each system. While TWIM-MS exhibited a calibration-dependent bias in comparison to DTIM-MS as reference, this was not observed for TIM-MS with DTIM-MS as reference.^18^ In a follow-up study, the authors probed the idea of using stable isotope-labeled steroid standards for internal monitoring of the CCS bias as well as an internal CCS correction to reduce systematic CCS bias.^19^ However, acquiring a sufficient number of stable isotope-labeled standards remains a cost-prohibitive limitation for this application.

In this regard, stable isotope labeled biomass materials are extremely attractive for use in metabolomics and lipidomics workflows, as they provide excellent coverage. Numerous materials are now available with the three main applications being (1) credentialing, (2) validation of isotopologue distributions, and (3) for normalization when quantifying.^20^ Recently, da Silva et al. proposed employing commercially available deuterium-labeled lipids as a quality control and/or system suitability material for IM experiments.^21^ However, when using deuterium-labeled lipid mixtures, the number of lipids is limited. To address this constraint, a stable isotope-labeled biomass, like the yeast strain *Komagataella phaffii* (also referred to as *Pichia pastoris*), can be employed.^22^ The uniformly (U)^13^C-labeled yeast extract is a well-established internal standard that covers a broad palette of U^13^C-labeled lipids that, up to this point, have been primarily employed for quantification workflows.^23−25^

In this work, we introduce MobiLipid, which utilizes a well-characterized fully U^13^C-labeled biomass for internal standardization of IM-MS lipidomics workflows to improve CCS quality control. The provided ^DT^CCS_N2_ library for U^13^C-labeled lipids can be utilized for internal CCS monitoring and correction without the requirement for additional external calibration data to be measured. It can be added to routine lipidomics workflows and requires only a low number of lipids per class to be detected for successful implementation. CCS monitoring and correction is automatized in form of an R Markdown.

## Experimental Section

### Sample Preparation

As samples, an unlabeled and a uniformly ^13^C-labeled ethanolic yeast (*Komagataella phaffii*) extract pellet^16^ were provided by Isotopic Solutions (Vienna, Austria). The yeast extract pellets were re-extracted using a two-phase lipidomics extraction based on methyl *tert*-butyl ether (MTBE) modified from Matyash et al.^26^ More details on the extraction procedure can be found in the Supporting Information. For the measurements, the extracts were dissolved in isopropanol and spiked with deuterated lipid standards (EquiSPLASH LIPIDOMIX Quantitative Mass Spec Internal Standard, Merck KGaA, Darmstadt, Germany). The extracts were measured in five different dilutions (Supporting Information).

### LC-IM-MS Methods

For LC-IM-MS measurements, two different IM technologies were used: (1) DTIM-MS using an Agilent 1290 Infinity II UPLC coupled to an Agilent 6560 IM-QTOFMS instrument equipped with an Agilent Dual Jet Stream ESI source and (2) TIM-MS using a Thermo Fisher Vanquish Horizon UPLC coupled to a Bruker timsTOF Pro instrument equipped with a Vacuum Insulated Probe Heated Electrospray Ionization (VIP-HESI). Reversed-phase (RP) LC separation using a C18 column with isopropanol-based gradient elution was performed according to Schoeny et al.^25^ (full LC method description is provided in Supporting Information).

LC-DTIM-MS measurements were acquired using the following ion source settings in positive mode: gas temperature of 250 °C and flow of 10 L/min (8 L/min for negative mode), nebulizer pressure of 40 psi (20 psi for negative mode), sheath gas temperature of 300 °C and flow of 12 L/min, capillary voltage of 3.5 kV (3.0 kV for negative mode), and nozzle voltage of 500 V. Data was acquired in MS1 mode with 4-bit multiplexing and a mass range of 50–1700 *m*/*z*. For IM separation, the following parameters were used: acquisition rate of 2 IM frames per second with 10 IM transients summed per frame, maximum drift time of 50 ms, trap fill time of 3200 μs, and trap release time of 150 μs. All data sets were CCS calibrated using the single-field method following established workflows.^6^

For LC-TIM-MS measurements, the instrument was mass and ^TIM^CCS_N2_ calibrated before measurement. Mass calibration was done with sodium formate clusters (10 mmol/L in 50:50 isopropanol/water (v/v)) using the High Precision Calibration (HPC) mode, and linear ^TIM^CCS_N2_ calibration was done using the Agilent ESI-L Tune Mix. Data was acquired using PASEF mode with a mass range of 100–1350 *m*/*z* and collision energy of 30 eV. The following ion source settings were used: capillary voltage of 4.5 kV, nebulizer gas pressure of 2.0 bar, drying gas flow rate of 8.0 L/min and temperature of 230 °C, and probe gas temperature of 300 °C. TIMS accumulation time was 100 ms with a TIMS ramp from 0.55 to 1.90 V·s/cm^2^.

### Data Preprocessing

LC-DTIM-MS data acquired with multiplexing was demultiplexed and smoothed using PNNL PreProcessor 4.1^27^ (2023.06.03) with the following parameters: For “Step 1: Data Compression and Interpolation” compress frames every 2 was chosen, and for “Step 2 (a): Multiplexed Data: Demux, Smooth, Spike Rem.” demultiplexing was done with chromatography/infusion (moving average) of 3 and minimum pulse coverage of 50% and a signal intensity lower threshold of 20 counts was set. DTIM-MS data was mass calibrated using the “lock masses” infused into the secondary nebulizer (purine and HP-921) in a postanalysis step using IM-MS Reprocessor (Agilent Technologies). Single-field CCS calibration was performed within IM-MS Browser 10.0 to yield linear calibration coefficients following an established procedure^6^ to apply to all corresponding measurement files within a single measurement sequence.

### Targeted Lipidomics Data Processing

All LC-IM-MS data were further processed using Skyline 23.1.0.268. After LC peak integration considering the equivalent carbon number (ECN) model^28,29^ for RP separation of lipids, an ion mobility library was created for each data file and ion mobility filtering was applied using the corresponding library with a resolving power of 50. The ion mobility dimension (i.e., drift time for DTIM-MS and 1/K_0_ for TIM-MS data) was manually inspected and if necessary corrected by setting an explicit ion mobility value under “Modify Custom Ion Precursor”. After exporting results, further data filtering, evaluation, and visualization was done in the R studio programming environment (R version 4.3.2 and RStudio 2023.09.1). The following filter criteria were applied: (1) low mass error of ≤5 ppm, (2) detection in at least 2 dilutions, (3) coelution of adducts (including matching of positive and negative ionization mode), and (4) a relative standard deviation <1% for CCS repeatability precision.

### Implementation of MobiLipid

MobiLipid is run in the R studio environment. Comprehensive documentation of required installations and how to run MobiLipid is provided at https://github.com/FelinaHildebrand/MobiLipid.

## Results and Discussion

### ^DT^CCS_N2_ Library for U^13^C-Labeled Lipids

**Figure 1:**
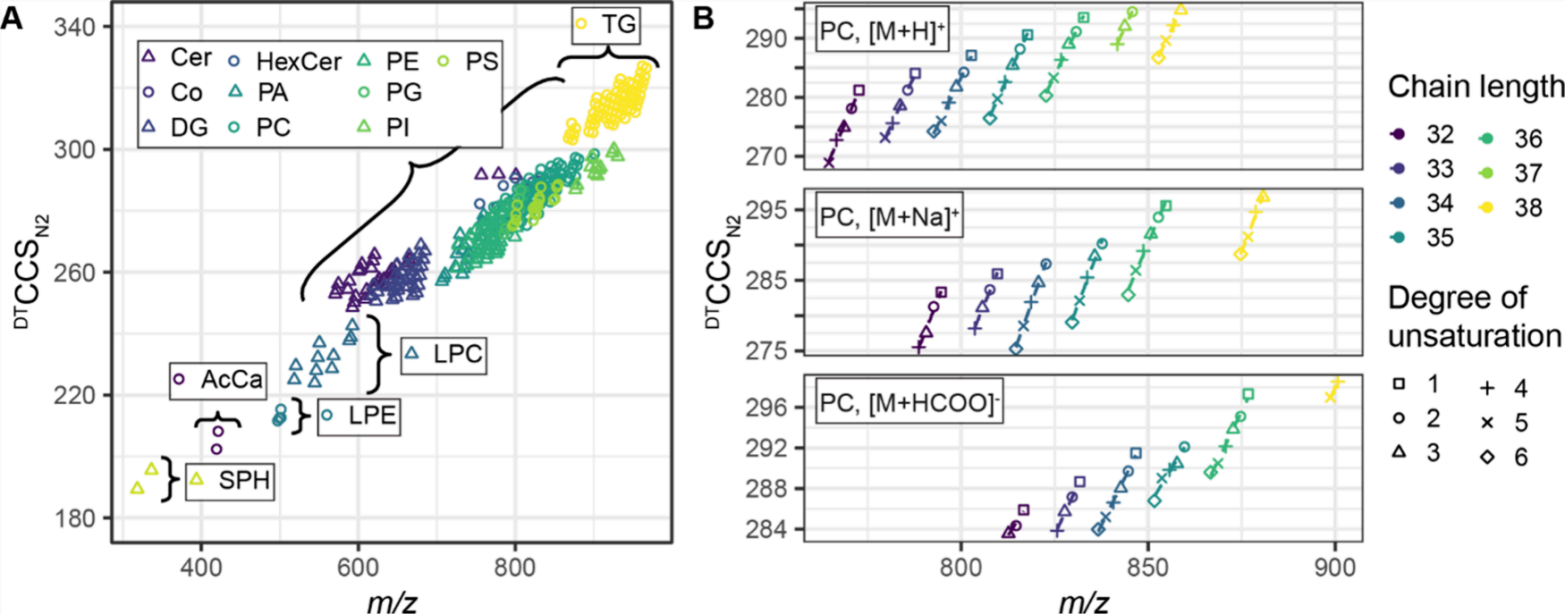
^DT^CCS_N2_ library for U^13^C-labeled yeast lipids. (A) *m*/*z* vs ^DT^CCS_N2_ values for all entries of the ^DT^CCS_N2_ library for U^13^C-labeled yeast lipids. The library contains 377 ^DT^CCS_N2_ values covering 15 lipid classes and 5 different adduct types ([M + H]^+^, [M + NH_4_]^+^, [M + Na]^+^, [M – H]^−^, and [M + HCOO]^−^). (B) ^DT^CCS_N2_ values for PCs, including their different adducts, represented analogously to the equivalent carbon number (ECN) model of retention times according to the fatty acyl chain length and degree of unsaturation.

In this work, we introduce a new ^DT^CCS_N2_ library for U^13^C-labeled lipids derived from a fully labeled yeast (*Komagataella phaffii*) extract.^22^ The library was established using DTIM-MS measurements as a reference method, providing a direct link to the current gold standard, i.e., the primary method of CCS determination, thus, ensuring the highest possible metrological trackability for LC-IM-MS.^6^ Specifically, LC-DTIM-MS measurements of a yeast extract dilution series in positive and negative ionization modes served for library generation. To ensure the quality of the library, only ^DT^CCS_N2_ values fulfilling the following criteria were included: (1) high mass accuracy (≤5 ppm error), (2) confirmed detection in at least two samples, (3) confirmed coelution of adducts, and (4) a CCS relative standard deviation <1%. Finally, the resulting ^DT^CCS_N2_ values were verified by plotting the entries for each lipid class versus the respective *m*/*z* values (Figure 1A). In analogy to the established equivalent carbon number (ECN) model,^28,29^ these plots ensure the quality of the data (Figures 1B and S1) as only CCS values following the same pattern, as would be expected for a chromatographic retention time, were accepted. When transferring the ECN model from the retention time to the CCS dimension, an increased fatty acyl chain length corresponds to a larger CCS value, and an increase in the degree of unsaturation corresponds to a smaller CCS value. After curation using these stringent criteria, the new MobiLipid library contains 377 ^DT^CCS_N2_ values for 162 U^13^C-labeled lipids detected at lipid species level across 15 lipid classes (AcCa, acylcarnitine; Cer, ceramide; Co, coenzyme; DG, diacylglycerol; HexCer, hexosylceramide; LPC, lysophosphatidylcholine; LPE, lysophosphatidylethanolamine; PA, phosphatidic acid; PC, phosphatidylcholine; PE, phosphatidylethanolamine; PG, phosphatidylglycerol; PI, phosphatidylinositol; PS, phosphatidylserine; SPH, sphingoid base; TG, triacylglycerol), including various adducts (i.e., [M + H]^+^, [M + NH_4_]^+^, [M + Na]^+^, [M – H]^−^, [M + HCOO]^−^) across a mass range of *m*/*z* 318.3501–966.9794 and ^DT^CCS_N2_ values are in the range 189.4–327.1 Å^2^ (Figure 1A). In the cases of lipid species detected as chromatographically resolved isomers, these are annotated by adding a number indicating the order of elution. The full ^DT^CCS_N2_ library for U^13^C-labeled yeast lipids is provided in the Supporting Information (Table S1).

### MobiLipid: Setting the Scene

**Figure 2:**
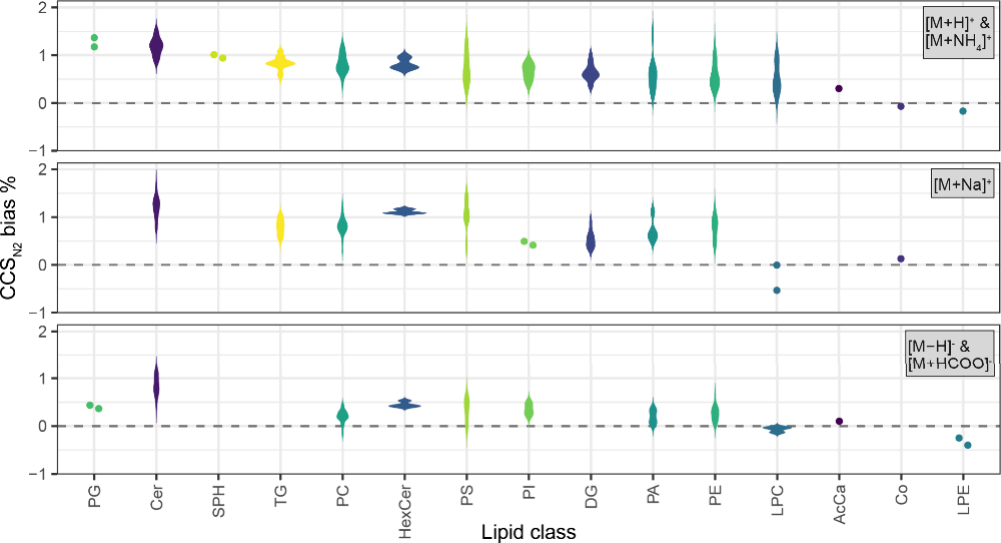
CCS_N2_ bias between ^DT^CCS_N2_ and ^TIM^CCS_N2_ of U^13^C-labeled lipids according to lipid class, whereby ^DT^CCS_N2_ is used as the reference point. Upper panel: bias for [M + H]^+^ and [M + NH_4_]^+^ adducts. Center panel: bias for [M + Na]^+^ adducts. Lower panel: bias for [M – H]^−^ and [M + HCOO]^−^ adducts.

To evaluate the use of the new U^13^C lipidomics library, experiments were carried out on two different IM-MS platforms. In the first step, LC-DTIM-MS and LC-TIM-MS measurements were performed using the U^13^C-labeled lipid yeast extract and its unlabeled version, together with a blend (50:50 v/v). Comparison of data for unlabeled (monoisotopic) and U^13^C-labeled lipids revealed a very small systematic shift of approximately 0.25% in ^DT^CCS_N2_ values that is consistent with reduced mass considerations observed with moderate resolution IM-MS (Supporting Information, Figure S5). Scrutinizing the ^DT^CCS_N2_ and ^TIM^CCS_N2_ values, the mean absolute CCS_N2_ bias of U^13^C-labeled lipids between the two IM technologies is 0.78% in positive ionization mode and 0.33% in negative ionization mode (using ^DT^CCS_N2_ values as a reference in each case). Figure 2 visualizes the CCS_N2_ bias between the two technologies for all U^13^C-labeled lipids categorized according to lipid class and the different adducts detected. The trend is similar for unlabeled lipids with a mean absolute CCS_N2_ bias of 0.73% in positive and 0.46% in negative ionization mode with no strong ion species-specific trends observed (Figure S6). Interestingly, the majority of the analyzed lipid classes exhibit a positive bias for ^TIM^CCS_N2_ values, indicating a systematic shift toward larger CCS_N2_ values when measuring TIM-MS compared to DTIM-MS, despite using the same external calibrant mixture (Figure 2). In terms of lipid classes, the bias ranged between −0.31 and 1.08% for U^13^C-labeled lipids, while the observed bias is in a similar range (−0.19 – 1.11%) for the unlabeled lipids. These findings are supported by previous studies.^17^ However, in the absence of a fundamental model that can correct these sources of bias between lipidomics data sets from different IM-MS technologies, we explored the use of the new MobiLipid U^13^C library as an internal standardization tool to address this.

### Application of MobiLipid: CCS Benchmarking and Internal CCS Standardization

The new MobiLipid ^DT^CCS_N2_ library for U^13^C-labeled lipids provides a means to achieve quality control by monitoring the potential CCS_N2_ bias of an IM-MS experiment. By spiking U^13^C-labeled lipids into measured samples and determination of the CCS_N2_ bias between derived CCS values and the ^DT^CCS_N2_ library, the extent of the bias can be assessed for each measured data file, providing a straightforward approach to achieving constant quality control using lipid class-specific labeled internal standards. As CCS values of small molecule ions determined by IM-MS technologies like TIM-MS and TWIM-MS are dependent on the external calibration of the instrument^19^ the source(s) of CCS bias introduced by the calibration process cannot be easily identified. As a result, correction approaches using new external calibrants are thwarted. In this work, we instead propose the use of a full palette of U^13^C-labeled lipids as an internal standard approach for LC-IM-MS lipidomics. This can deliver significant advantages for maintaining data quality across different IM-MS platforms and for dealing with annotation of diverse lipid classes across different lipidomics applications. The mathematics behind the internal standardization in MobiLipid are linear correction functions, based on the derived CCS_N2_ of U^13^C-labeled lipids and the deployed reference ^DT^CCS_N2_ values, following the approach of Deschamps et al.^14^ who used linear CCS correction function for phospholipids utilizing an externally measured lipid standard. The functions require a minimum of three labeled lipids as input for each lipid class-adduct combination. Due to the library composition, the correction procedure can cover the following 10 lipid classes: Cer, DG, HexCer, LPC, PA, PC, PE, PI, PS, and TG. Finally, the CCS_N2_ value of all lipids within a lipid class-adduct combination are corrected by inserting the derived CCS_N2_ value in the correction functions.

The correction procedure in MobiLipid is automated using R Markdown and available on GitHub (https://github.com/FelinaHildebrand/MobiLipid). After generating up to 100 distinct correction functions employing 3–6 lipids for linear regression, depending on the overall count of labeled lipids within a lipid class–adduct combination, corrected CCS_N2_ values are derived for all lipids within the lipid class–adduct combination, irrespective of their labeling status. Additionally, the mean bias before and after correction relative to library ^DT^CCS_N2_ values for labeled lipids is computed and reported.

To illustrate the applicability of MobiLipid, we applied the R Markdown tool to the mean values of all TIM-MS data acquired for this work. Linear regression for the generation of correction function was based on mean ^TIM^CCS_N2_ values and ^DT^CCS_N2_ library values. Subsequently, applying all generated functions facilitated the correction of ^TIM^CCS_N2_ values for both unlabeled and U^13^C-labeled lipids. The exemplary generated results, including all correction functions, corrected CCS_N2_ values, and CCS_N2_ bias before and after correction can be accessed in the Supporting Information (Tables S2–S7). Overall, it becomes evident that for all 10 lipid classes included in the CCS correction procedure of MobiLipid the average bias between ^TIM^CCS_N2_ and ^DT^CCS_N2_ tends to approach zero when applying lipid class- and adduct-specific correction functions, irrespective of the number of lipids utilized to establish the function (Figure S7). In total, the CCS_N2_ values of 297 lipids from 10 different lipid classes, irrespective of their labeling status, were corrected.

**Figure 3:**
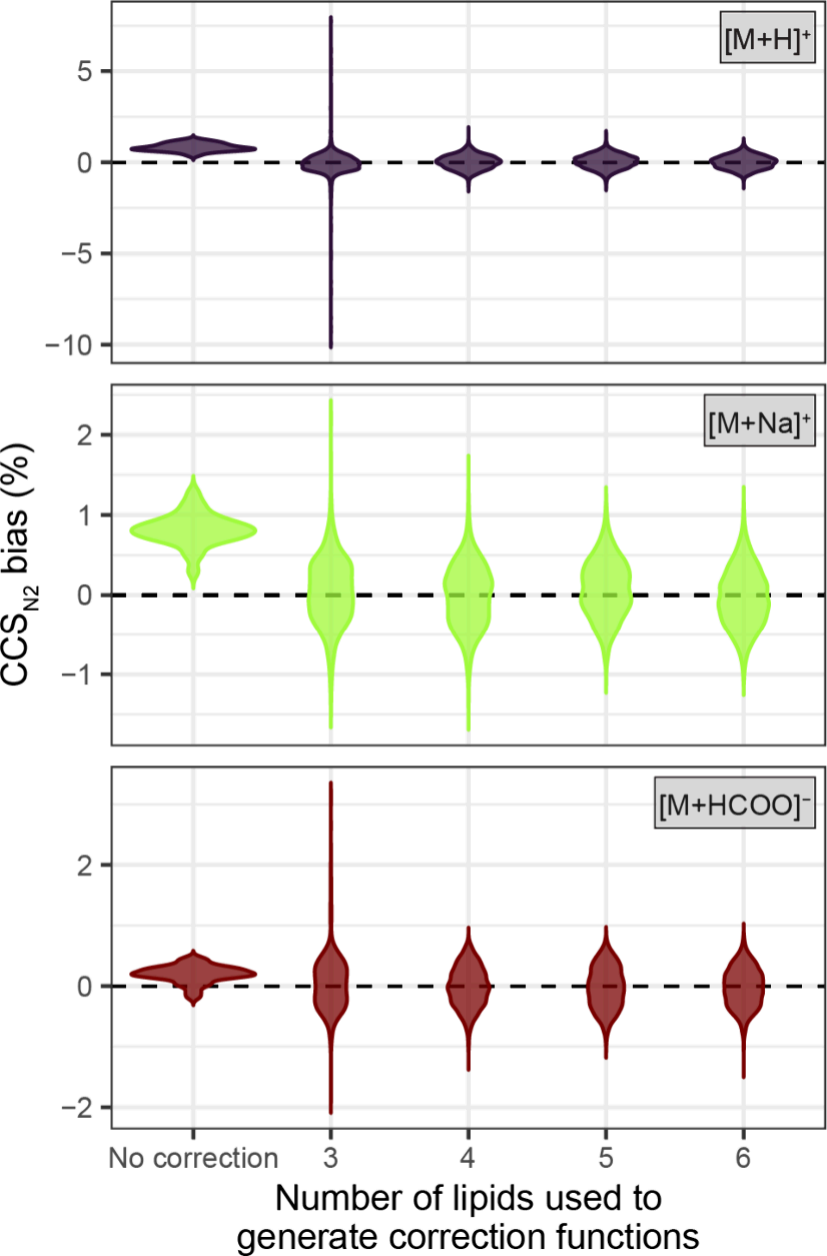
CCS_N2_ bias (%) between ^DT^CCS_N2_ and ^TIM^CCS_N2_ of PCs (^DT^CCS_N2_ as a reference for calculation) before and after CCS correction using MobiLipid. For the correction, up to 100 distinct correction functions using 3–6 lipids within a lipid class-adduct combination were generated, and the ^TIM^CCS_N2_ value of each lipid was corrected with all functions. The CCS_N2_ bias distribution is plotted, depending on the number of lipids used to generate the correction functions for all three adducts detected for PCs.

Figure 3 illustrates the CCS_N2_ bias before and after correction exemplary of PCs for which a broad coverage was achieved in this work. The majority of the computed correction functions exhibit good performance, as evidenced by the effective reduction of CCS_N2_ bias between corrected CCS_N2_ values and ^DT^CCS_N2_ library values. Using a higher number of U^13^C-labeled lipids to compute the correction functions leads to a more robust correction. However, a few correction functions exhibit subpar performance, which leads to a wider distribution of the CCS_N2_ bias. While the required minimum of three U^13^C lipids yielded a good correction performance for most of the correction functions, their performance should be controlled before reporting corrected CCS_N2_ values. For the example of PCs, this can be observed especially when sampling different combinations of only 3 U^13^C lipids detected as [M + H]^+^ adducts for the generation of lipid class- and adduct-specific correction functions. In addition to the plot shown in Figure 3, MobiLipid plots the CCS bias distribution for each correction function (corresponding to a unique set of U^13^C lipids) separately (Figure S8). This plot allows the user to manually inspect the performance of each correction function. For the exemplary data it becomes apparent that only 3 of the 100 generated functions using three U^13^C labeled lipids lead to an unsatisfactory distribution of CCS bias, i.e., functions 31, 93, and 98 (Figure S8). These three functions exhibit the highest deviation from a slope of 1 (i.e., 0.01, 0.46, and 2.42, Table S3), indicating a poor linear relationship which leads to subpar CCS correction results. MobiLipid allows user-level confirmation of the quality of correction functions for all 10 lipid classes utilized in the CCS correction procedure. The correction procedure within MobiLipid shows an excellent performance for TIM-MS data. However, in the case of TWIM-based systems, which have different external calibration characteristics, it might require some additional scrutiny.

## Conclusion

MobiLipid is a fully automated R Markdown tool utilizing a newly established ^DT^CCS_N2_ library for U^13^C-labeled lipids to internally assess CCS_N2_ bias in IM-MS lipidomics workflows and allow CCS correction without the need for additional external calibration procedures. As MobiLipid is based on an internal standardization strategy, it is applicable to lipidomics workflows performed on different IM-MS technologies. The labeled internal standard can be integrated into IM-MS lipidomics workflows targeting both exploratory and quantitative analysis. The incorporation of the U^13^C-labeled internal standard provides a 2-fold benefit, as it facilitates CCS quality control utilizing MobiLipid as well as ensuring accurate lipid quantification, as shown elsewhere.^23−25^ Furthermore, the MobiLipid strategy could be further extended to other isotopically labeled lipid materials using the same internal standardization approach elaborated here.

## Data Availability

Raw data for LC-DTIM-MS and LC-TIM-MS is made available on MassIVE (ftp://massive.ucsd.edu/v07/MSV000094211).

## Supporting Information Available

Supplementary Experimental Section, including detailed sample preparation workflow, LC method, settings for data processing, and CCS bias calculation; Supplementary figures, including equivalent carbon number model for CCS values, CCS bias before and after CCS correction using MobiLipid, CCS bias for distinct correction functions, CCS bias-dependent on modified CCS, and CCS bias between unlabeled and U^13^C-labeled lipids (PDF)
Supplementary tables, including ^DT^CCS_N2_ library for U^13^C-labeled lipids, exemplary output tables of MobiLipid, and results for LC-DTIM-MS and LC-TIM-MS measurements (XLSX)
