# Supplementary material for: MobiLipid: A Tool for Enhancing CCS Quality Control of Ion Mobility–Mass Spectrometry Lipidomics by Internal Standardization
Source: Anal Chem. 2024 May 2;96(19):7380–5. doi: 10.1021/acs.analchem.4c01253 (PMC11099887; doi:10.1021/acs.analchem.4c01253)
Supplement: Supplementary file 1 — ac4c01253_si_001.pdf [file ac4c01253_si_001.pdf]

Supporting Information

MobiLipid: A Tool for Enhancing CCS Quality Control of IM-MS Lipidomics by Internal Standardization

Felina Hildebrand[a,b], Gunda Koellensperger[a,c*], Tim Causon[d*]

[a] *Department of Analytical Chemistry, Faculty of Chemistry, University of Vienna, Waehringer Str. 38, 1090 Vienna, Austria*

[b] *Vienna Doctoral School in Chemistry (DoSChem), University of Vienna, Vienna, Austria*

[c] *Vienna Metabolomics Center (VIME), University of Vienna, Althanstr. 14, 1090 Vienna, Austria*

[d] *BOKU University, Department of Chemistry, Institute of Analytical Chemistry, Muthgasse 18, 1190 Vienna, Austria*

* Corresponding authors: Gunda Koellensperger: gunda.koellensperger@univie.ac.at;
Tim Causon: tim.causon@boku.ac.at

Table of Contents

Supplementary Experimental Section S2

Solvents and Standards S2

Sample preparation S2

LC method S3

Skyline settings for data processing S3

Calculation of CCS bias S3

Supplementary Figures S4

Figure S1 .... S5

Figure S2 .... S6

Figure S3 .... S7

Figure S4 .... S8

Figure S5 .... S9

Figure S6 .... S9

Figure S7 .... S10

Figure S8 .... S11

References S12

Supplementary Experimental Section

Solvents and Standards

All solvents (acetonitrile, water, isopropanol, methanol, and methyl tert-butyl ether) were ordered at Honeywell (Vienna, Austria) with LC-MS grade. The eluent additives ammonium formate and formic acid were purchased from Sigma Aldrich (Vienna, Austria) and VWR International (Vienna, Austria), respectively.

The deuterated lipid standard mix, EquiSPLASH™ LIPIDOMIX® Quantitative Mass Spec Internal Standard, was acquired from Merck KGaA (Darmstadt, Germany).

Sample preparation

The ethanolic yeast extract pellets[1] were re-extracted using the following extraction procedure[2]:

To each extract, 600 µL MTBE and 200 µL methanol (MeOH) were added and vigorously mixed to dissolve the pellets. After shaking for 15 min at 12°C and 600 rpm, 200 µL water was added before extracts were again mixed vigorously and then centrifuged (10 min at 12°C and 4500 rpm). After completing the phase separation process, the upper organic phase was transferred into a 2-mL microcentrifuge tube and dried under vacuum (Genevac EZ-2 Personal Evaporator). Extracts were dissolved in 2 mL isopropanol (IPA) by sonication for 1 min. The extracts (unlabeled and U^{13}C-labeled) were each aliquoted into 10 µL aliquots. Additionally, mixed aliquots were prepared by combining 10 µL of each extract. All aliquots were dried again and stored at -80°C. For the measurements, aliquots were dissolved according to Table 1 to obtain five dilutions for each extract (unlabeled, U^{13}C labeled, and 50:50 mix of both extracts). For the preparation EquiSPLASH™ was diluted 1:15 with IPA.

Table 1: Preparation of yeast extracts for LC-IM-MS measurements. Each solution was prepared for 3 different yeast extracts (unlabeled, $U^{13}C$ labeled, and 50:50 mix of both extracts).

Solution	Preparation
1	Add to dried yeast extract:<br>45 µL IPA<br>5 µL 1:15 EquiSPLASH™
2	Add to dried yeast extract:<br>95µL IPA<br>5 µL 1:15 EquiSPLASH™
3	Add to dried yeast extract:<br>195 µL IPA<br>5 µL 1:15 EquiSPLASH™
4	1:5 dilution of solution 2
5	1:5 dilution of solution 3

LC method

LC separation was done following the method by Schoeny *et al.*[3]. Briefly, an ACQUITY UPLC HSS T3 column (2.1 x 150 mm, 1.8 µm, Waters) along with an ACQUITY UPLC HSS T3 VanGuard Pre-column (2.1 x 5 mm, 1.8 µm, Waters) were used for the reversed-phase LC separation. The mobile phases were ACN/water (3:2, v/v) as eluent A and IPA/ACN (9:1, v/v) as eluent B, both containing 10 mmol/L ammonium formate and 0.1% formic acid. The flow rate was 0.25 mL min^{-1} and the following gradient was run: 0 – 8 min 55% B to 65% B, 8 – 13 min ramp to 85% B, 13 – 15 min ramp to 100% B, 15 – 20 min 100% B, and 20 – 23 min re-equilibration at 55% B. The autosampler temperature and the column temperature were consistently maintained at 10°C and 40°C, respectively and the injection volume was 5 µL.

Skyline settings for data processing

Under Transition Settings “Full-Scan” the following parameters were used for “MS1 filtering”: Isotope peaks included was set to count with Peaks 1 and Precursors mass analyzer was set to TOF with Resolving power of 20 000 for LC-DTIM-MS and 30 000 for LC-TIM-MS data. Additionally, “Use high-selectivity extraction” was selected.

Calculation of CCS bias

The CCS bias between $^{TIM}CCS_{N2}$ and $^{DT}CCS_{N2}$ was calculated with $^{DT}CCS_{N2}$ values as references using the following equation:

$$CCS\ bias\ (\%) = \frac{^{TIM}CCS_{N2} - {}^{DT}CCS_{N2}}{^{DT}CCS_{N2}} \quad \text{(Eq. 1)}$$

The CCS bias between $^{DT}CCS_{N2}$ values for unlabeled lipids and $^{DT}CCS_{N2}$ values for $U^{13}C$ labeled lipids was calculated with $^{DT}CCS_{N2}$ values for unlabeled lipids as references using the following equation:

$$CCS\ bias\ (\%) = \frac{^{U^{13}C\ labeled,DT}CCS_{N2} - {}^{unlabeled,DT}CCS_{N2}}{^{unlabeled,DT}CCS_{N2}} \quad \text{(Eq. 2)}$$

Supplementary Figures

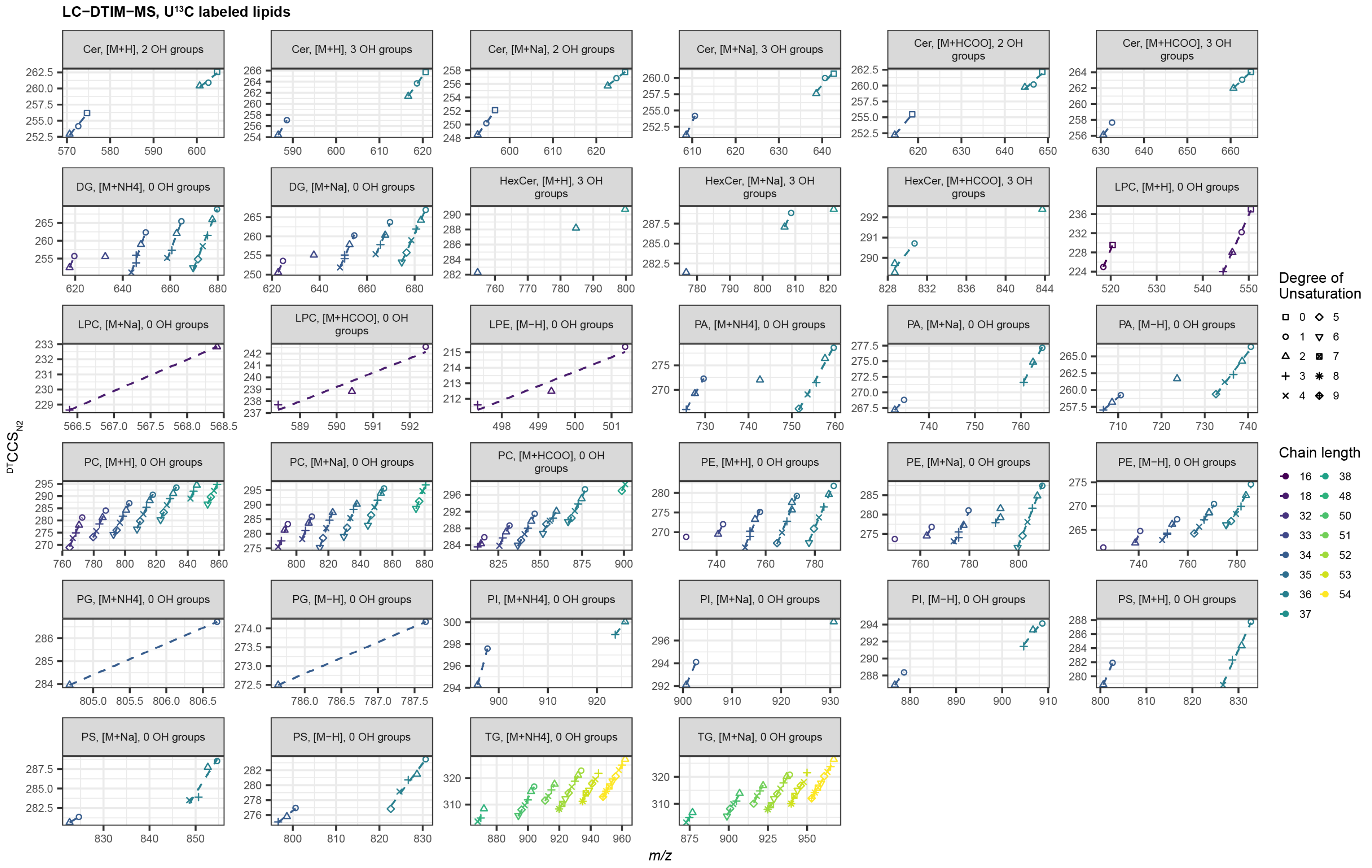

Figure S1. $^{DT}CCS_{N2}$ values for U^{13}C labeled lipids represented analogously to the equivalent carbon number (ECN)[4,5] model of retention times according to the fatty acyl chain length and degree of unsaturation. Only groups (Lipid class, adduct, number of OH group) which contain more than 1 lipid are plotted.

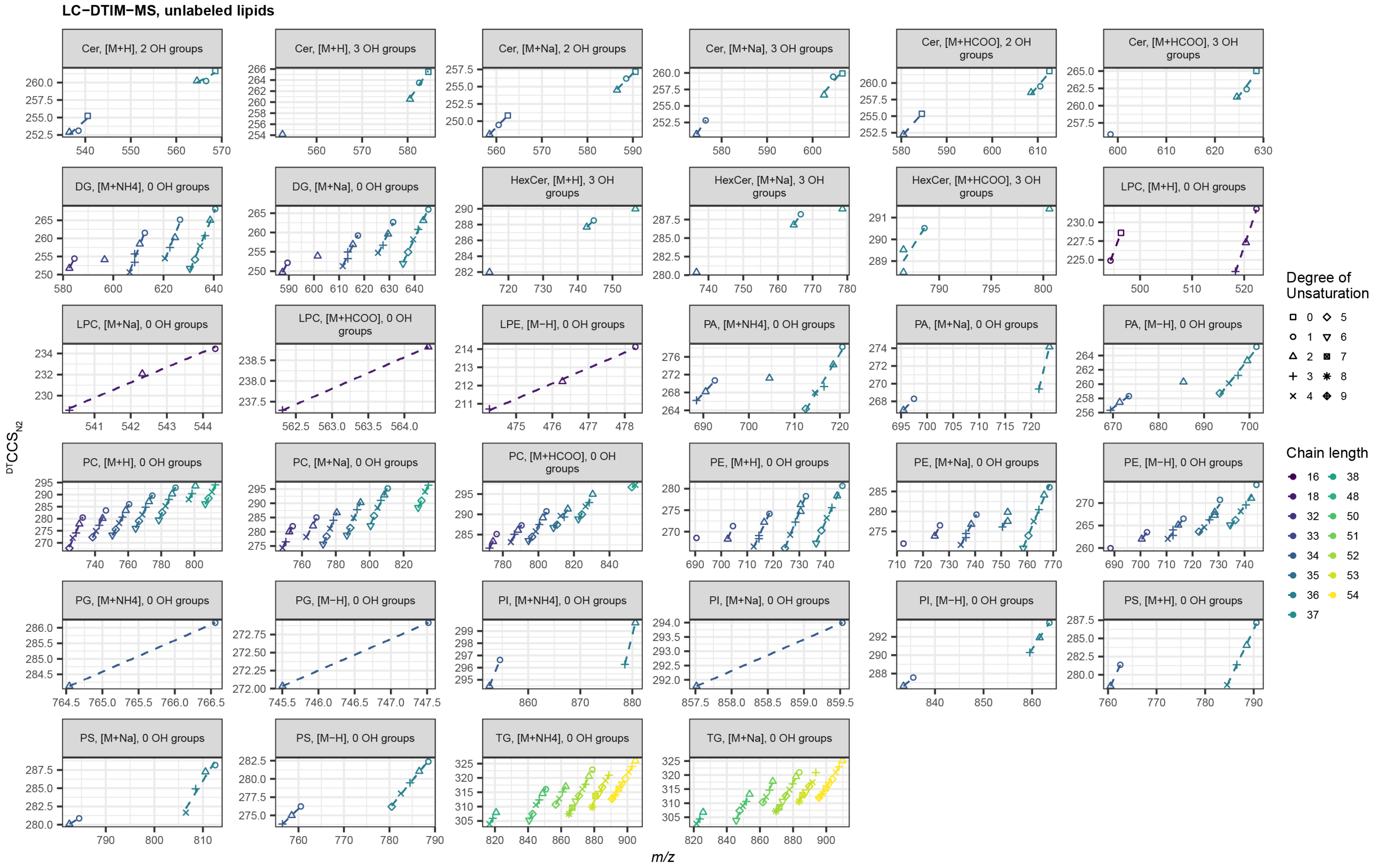

Figure S2. $^{DT}CCS_{N2}$ values for unlabeled lipids represented analogously to the equivalent carbon number (ECN)[4,5] model of retention times according to the fatty acyl chain length and degree of unsaturation. Only groups (lipid class, adduct, number of OH group) which contain more than 1 lipid are plotted.

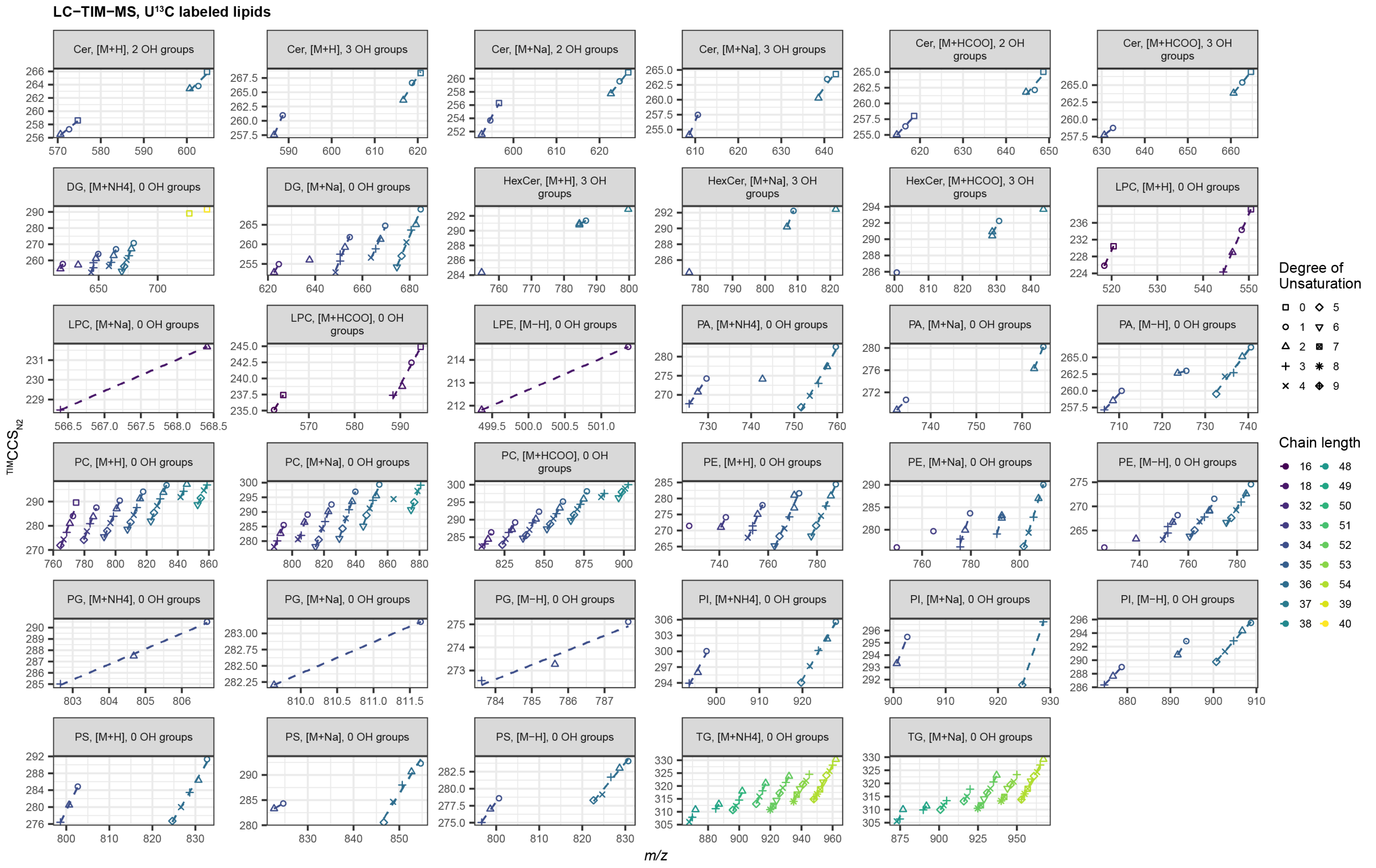

Figure S3. $^{TIM}CCS_{N2}$ values for $U^{13}C$ labeled lipids represented analogously to the equivalent carbon number (ECN)[4,5] model of retention times according to the fatty acyl chain length and degree of unsaturation. Only groups (lipid class, adduct, number of OH group) which contain more than 1 lipid are plotted.

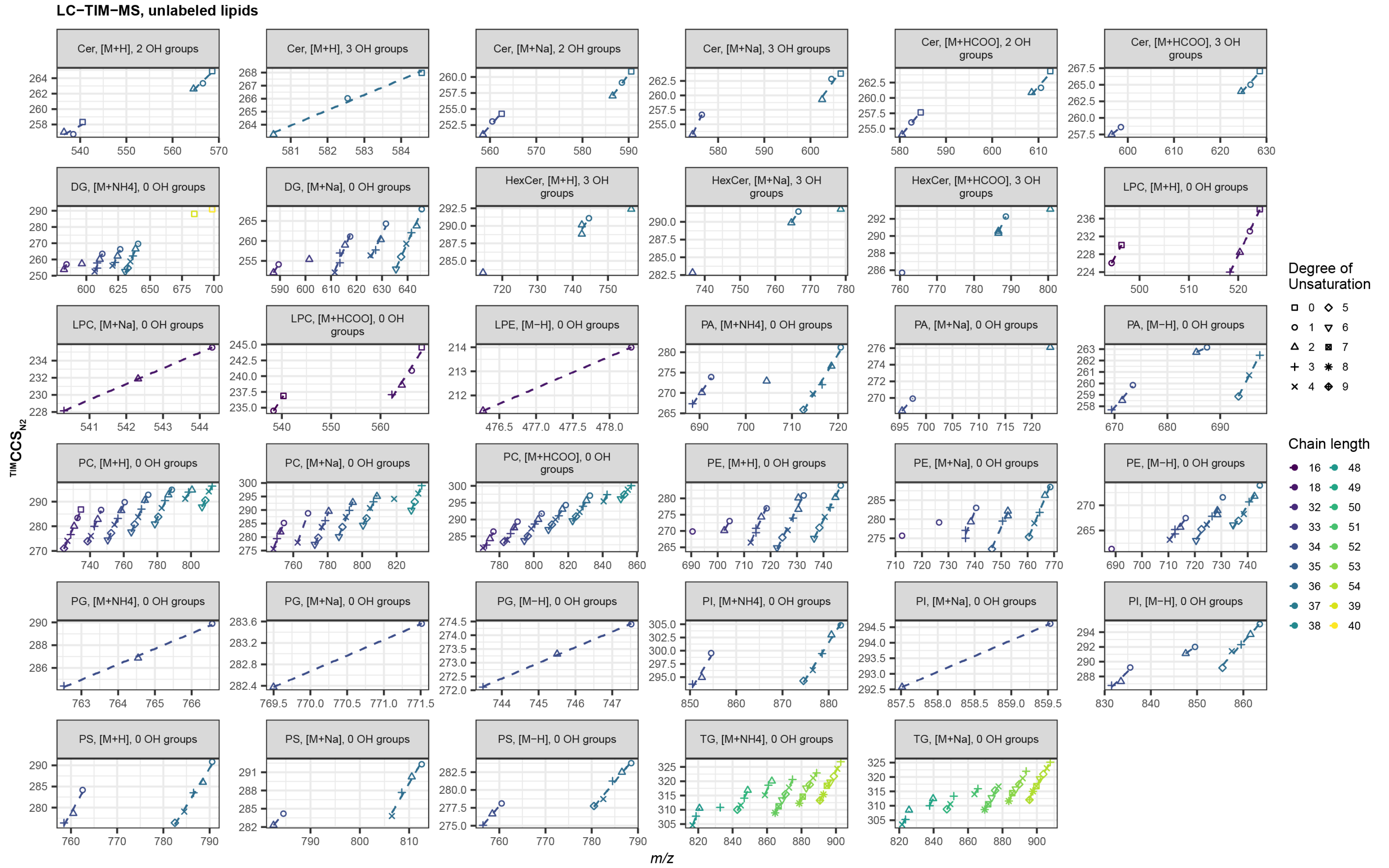

Figure S4. $^{TIM}CCS_{N2}$ values for unlabeled lipids represented analogously to the equivalent carbon number (ECN)[4,5] model of retention times according to the fatty acyl chain length and degree of unsaturation. Only groups (lipid class, adduct, number of OH group) which contain more than 1 lipid are plotted.

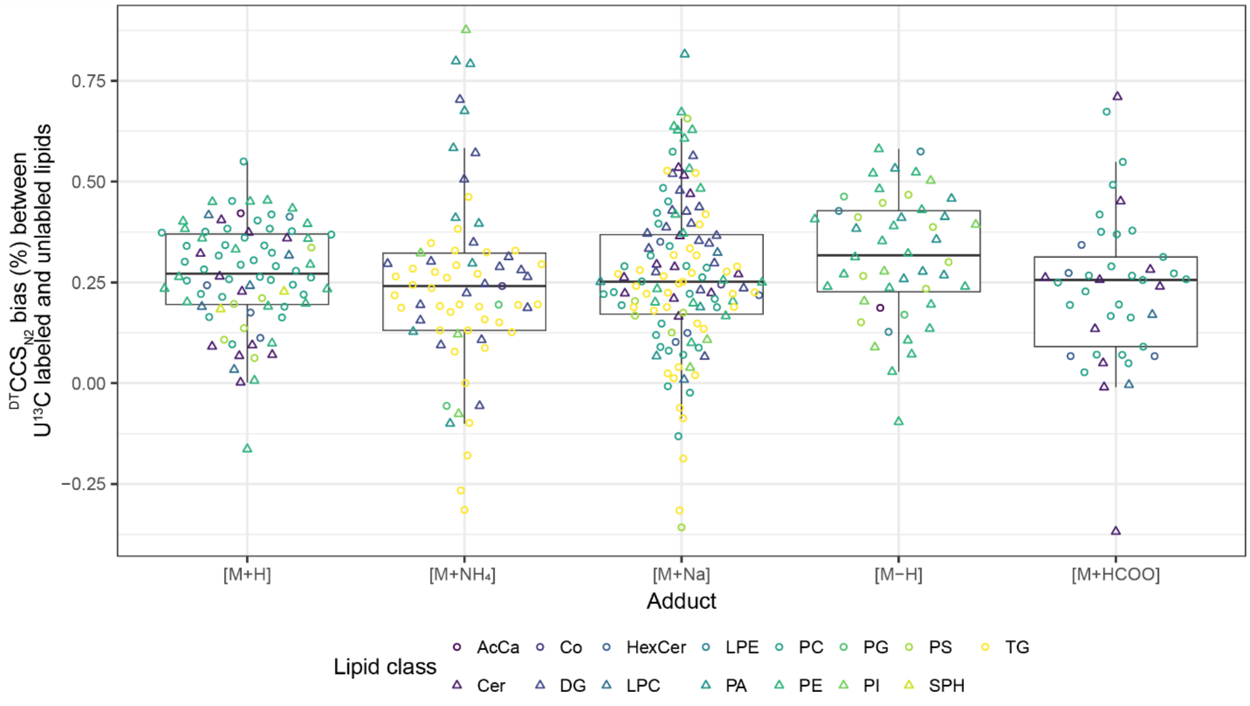

Figure S5. $^{DT}CCS_{N2}$ bias between unlabeled and U[13]C labeled lipids ($^{DT}CCS_{N2}$ of unlabeled lipids as reference for calculation, Eq. 2).

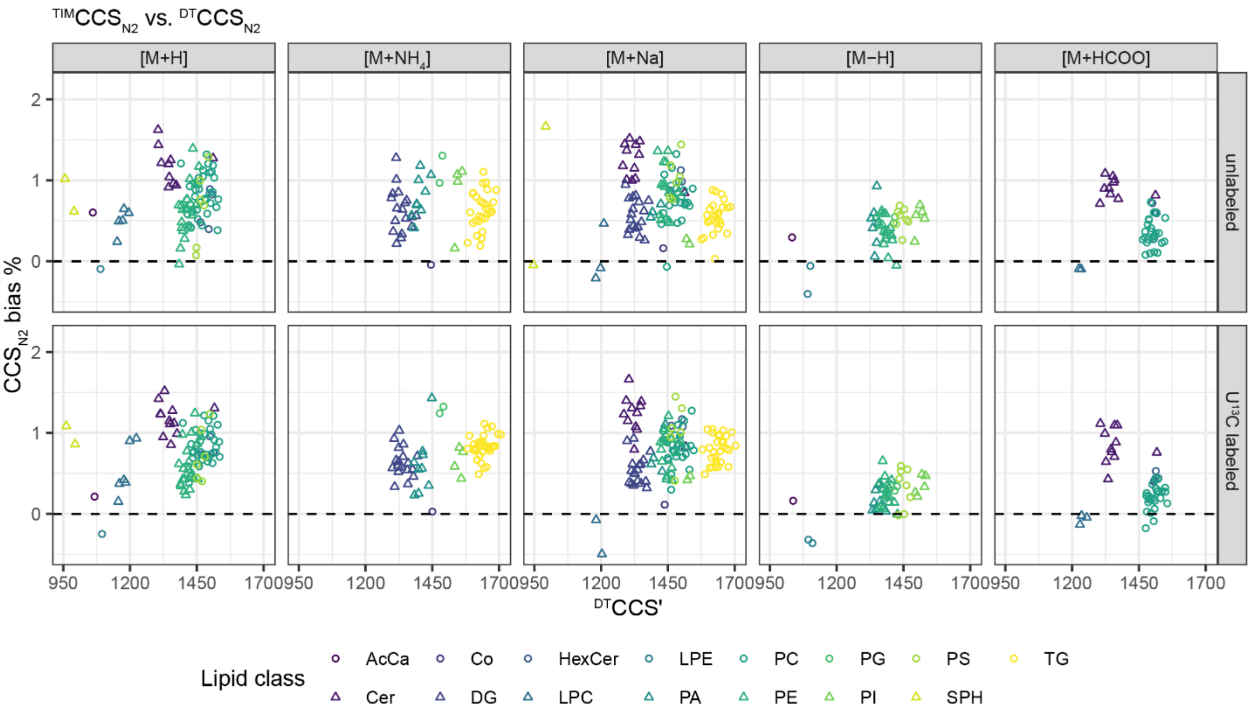

Figure S6. CCS_{N2} bias between $^{DT}CCS_{N2}$ and $^{TIM}CCS_{N2}$ of unlabeled and U[13]C labeled lipids ($^{DT}CCS_{N2}$ as reference for bias calculation, Eq. 1) dependent on $^{DT}CCS'$ (modified CCS) .

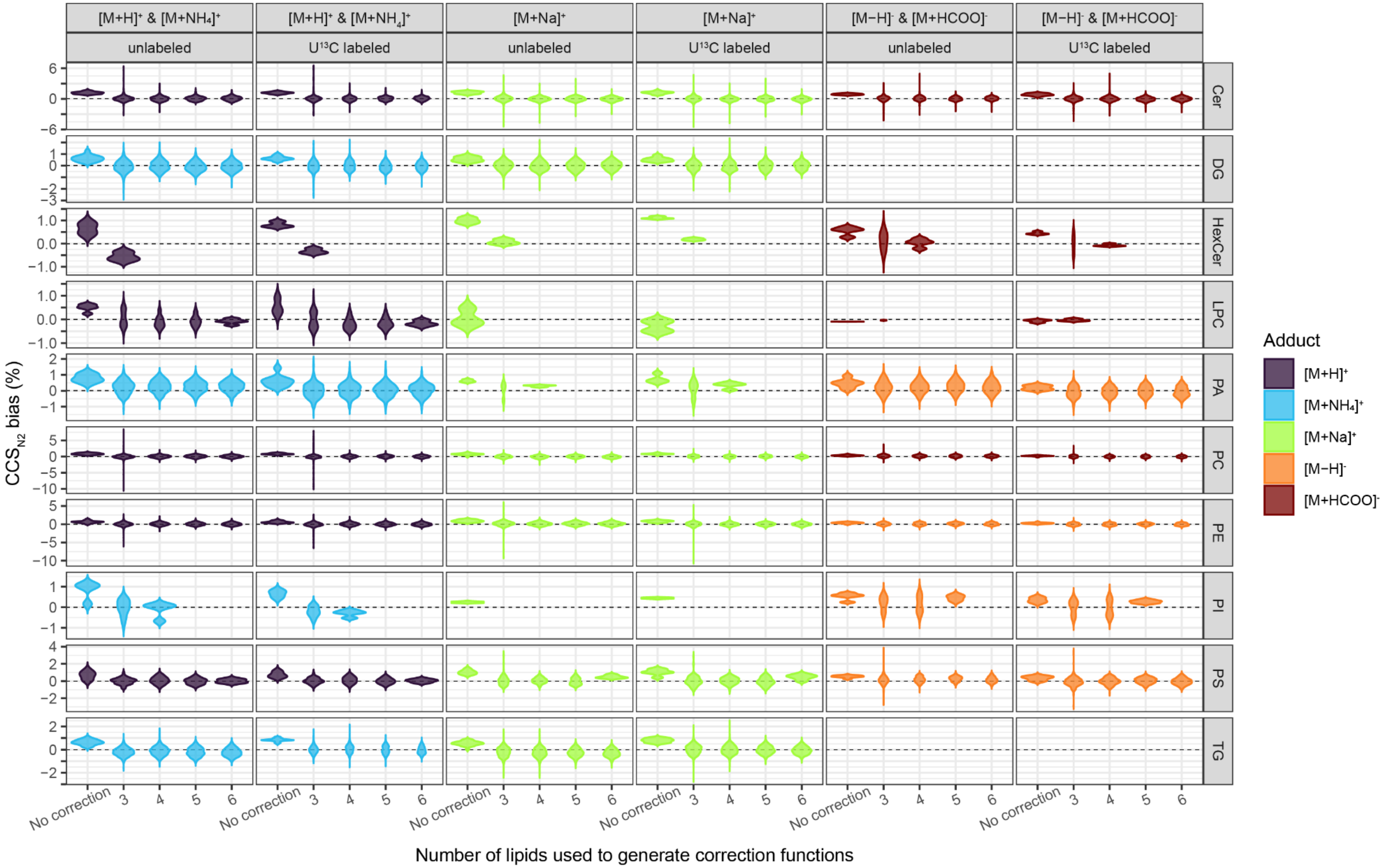

Figure S7. CCS_{N2} bias (%) between $^{DT}CCS_{N2}$ and $^{TIM}CCS_{N2}$ ($^{DT}CCS_{N2}$ as reference for calculation, Eq. 1) before and after CCS correction using MobiLipid. For the correction, up to distinct 100 correction functions using 3 to 6 lipids within a lipid class-adduct combination were generated and the $^{TIM}CCS_{N2}$ value of each lipid was corrected with all functions. The CCS_{N2} bias distribution is plotted dependent on the number of lipids used to generate the correction functions.

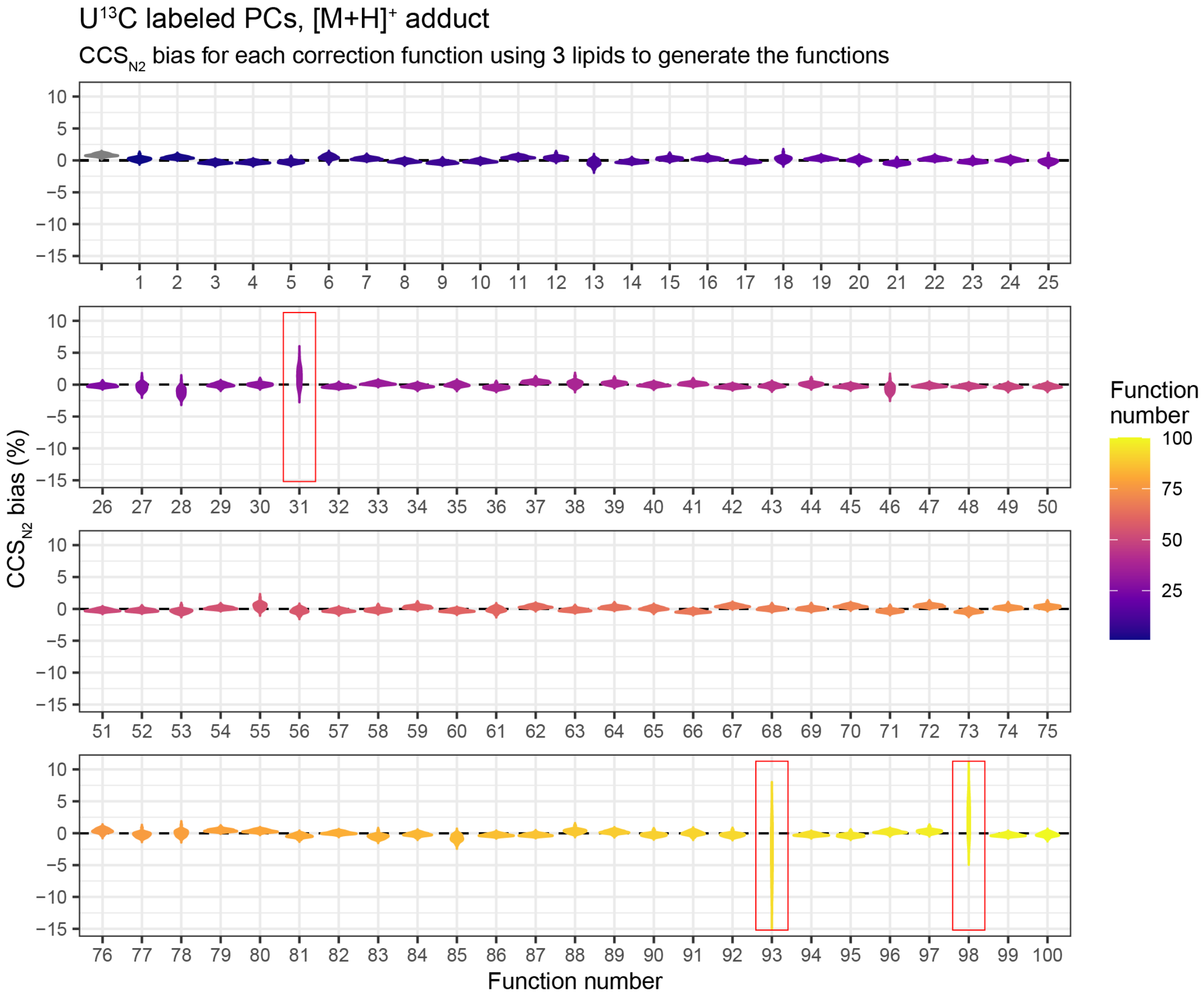

Figure S8. CCS_{N2} bias (%) between $^{DT}CCS_{N2}$ and $^{TIM}CCS_{N2}$ of PCs as $[M+H]^+$ adduct ($^{DT}CCS_{N2}$ as reference for calculation) before and after CCS correction with 100 distinct correction functions using each 3 lipids for linear regression. The CCS_{N2} bias distribution is plotted against each correction function. CCS correction functions with subpar performance leading to a wide CCS bias distribution are marked with red rectangles.

References

(1) Neubauer, S.; Haberhauer-Troyer, C.; Klavins, K.; Russmayer, H.; Steiger, M. G.; Gasser, B.; Sauer, M.; Mattanovich, D.; Hann, S.; Koellensperger, G. U ^{13}C Cell Extract of P Ichia Pastoris – a Powerful Tool for Evaluation of Sample Preparation in Metabolomics. *J. Sep. Sci.* **2012**, *35* (22), 3091–3105. https://doi.org/10.1002/jssc.201200447.

(2) Matyash, V.; Liebisch, G.; Kurzchalia, T. V.; Shevchenko, A.; Schwudke, D. Lipid Extraction by Methyl- *Tert* -Butyl Ether for High-Throughput Lipidomics. *J. Lipid Res.* **2008**, *49* (5), 1137–1146. https://doi.org/10.1194/jlr.D700041-JLR200.

(3) Schoeny, H.; Rampler, E.; El Abiead, Y.; Hildebrand, F.; Zach, O.; Hermann, G.; Koellensperger, G. A Combined Flow Injection/Reversed-Phase Chromatography–High-Resolution Mass Spectrometry Workflow for Accurate Absolute Lipid Quantification with ^{13}C Internal Standards. *Analyst* **2021**, *146* (8), 2591–2599. https://doi.org/10.1039/D0AN02443K.

(4) Lísa, M.; Holčapek, M. Triacylglycerols Profiling in Plant Oils Important in Food Industry, Dietetics and Cosmetics Using High-Performance Liquid Chromatography–Atmospheric Pressure Chemical Ionization Mass Spectrometry. *J. Chromatogr. A* **2008**, *1198–1199*, 115–130. https://doi.org/10.1016/j.chroma.2008.05.037.

(5) Dugo, P.; Cacciola, F.; Kumm, T.; Dugo, G.; Mondello, L. Comprehensive Multidimensional Liquid Chromatography: Theory and Applications. *J. Chromatogr. A* **2008**, *1184* (1–2), 353–368. https://doi.org/10.1016/j.chroma.2007.06.074.
